# Supplementary material for: Was facial width-to-height ratio subject to sexual selection pressures? A life course approach
Source: PLoS One. 2021 Mar 12;16(3):e0240284. doi: 10.1371/journal.pone.0240284 (PMC7954343; doi:10.1371/journal.pone.0240284)
Supplement: S1 Table — (DOCX) [file pone.0240284.s002.docx]

S1 Table. Multicollinearity in fWHR measures [partial correlations for females (upper triangle) and males (lower triangle), controlling for age (3D sample)].

|  | **fWHR*nasion***  (nasion to labiale superius) | **fWHR*stomion***  (nasion to stomion) | **fWHR*lower***  (nasion to bottom of chin) | **Cheekbone Prominence** | **BMI** |
| --- | --- | --- | --- | --- | --- |
| **fWHR*nasion***  (nasion to labiale superius) | -- | .96*** | .78*** | .07* | .19*** |
| **fWHR*stomion***  (nasion to stomion) | .96*** | -- | .85*** | .09** | .21*** |
| **fWHR*lower***  (nasion to bottom of chin) | .79*** | .85*** | -- | .12*** | .09** |
| **Cheekbone Prominence** | .15*** | .17*** | .18*** | -- | -.08** |
| **BMI** | .25*** | .26*** | .17*** | -.01 |  |

**Note**. Significance (two-tailed)

†*P* < 0.10, **P* < 0.05, ***P* < 0.01, ****P* ≤ 0.001.
