# Supplementary material for: Was facial width-to-height ratio subject to sexual selection pressures? A life course approach
Source: PLoS One. 2021 Mar 12;16(3):e0240284. doi: 10.1371/journal.pone.0240284 (PMC7954343; doi:10.1371/journal.pone.0240284)
Supplement: S2 Table — (DOCX) [file pone.0240284.s003.docx]

S2 Table. Multicollinearity in fWHR measures [partial correlations for females (upper triangle) and males (lower triangle), controlling for age (2D sample)].

|  | **fWHR*nasion***  (nasion to labiale superius) | **fWHR**  ***brow***  (brow to labiale superius) | **fWHR**  ***stomion***  (nasion to stomion) | **fWHR*lower***  (nasion to bottom of chin) | **Cheekbone Prominence** | **BMI** |
| --- | --- | --- | --- | --- | --- | --- |
| **fWHR*nasion***  (nasion to labiale superius) | -- | .78*** | .40*** | .39** | -.39*** | .32** |
| **fWHR*brow***  (brow to labiale superius) | .82*** | -- | .78*** | .51*** | -.20† | .42*** |
| **fWHR*stomion***  (nasion to stomion) | .71*** | .92*** | -- | .63*** | -.07 | .34** |
| **fWHR*lower***  (nasion to bottom of chin) | .20† | .29** | .38*** | -- | .27* | .06 |
| **Cheekbone Prominence** | -.40*** | -.15 | -.12 | .57*** | -- | -.15 |
| **BMI** | .04 | .18 | .18 | -.54*** | -.37*** | -- |

**Note**. Significance (two-tailed)

†*P* < 0.10, **P* < 0.05, ***P* < 0.01, ****P* ≤ 0.001.
